# Augmenting large language models to predict social determinants of mental health in opioid use disorder using patient clinical notes

**DOI:** 10.1093/jamiaopen/ooaf142

**Published:** 2025-11-27

**Authors:** Madhavi Pagare, Deva Sai Kumar Bheesetti, Inyene Essien-Aleksi, Mohammad Arif Ul Alam

**Affiliations:** Cognitive Ubiquitous Computing and System (CUBICS) Lab, University of Massachusetts Lowell, Lowell, MA 01854, United States; Cognitive Ubiquitous Computing and System (CUBICS) Lab, University of Massachusetts Lowell, Lowell, MA 01854, United States; Merrimack College, North Andover, MA, 01845, United States; Cognitive Ubiquitous Computing and System (CUBICS) Lab, University of Massachusetts Lowell, Lowell, MA 01854, United States; Department of Medicine University of Massachusetts Chan Medical School, Worcester, MA, 01655, United States; National Institute on Aging, National Institute of Health, Bethesda, MD, 20892, United States

**Keywords:** social determinants of mental health (SDOMH), opioid use disorder, opioid-related Outcomes, Human-in-the-Loop LLM Interaction for Annotation (HLLIA), Multilevel Hierarchical Clinical-Longformer Embeddings (MHCLE), natural language processing (NLP)

## Abstract

**Objective:**

Identifying social determinants of mental health (SDOMH) in patients with opioid use disorder (OUD) is crucial for estimating risk and enabling early intervention. Extracting such data from unstructured clinical notes is challenging due to annotation complexity and requires advanced natural language processing (NLP) techniques. We propose the Human-in-the-Loop Large Language Model Interaction for Annotation (HLLIA) framework, combined with a Multilevel Hierarchical Clinical-Longformer Embedding (MHCLE) algorithm, to annotate and predict SDOMH variables.

**Materials and Methods:**

We utilized 2636 annotated discharge summaries from the Medical Information Mart for Intensive Care (MIMIC-IV) dataset. High-quality annotations were ensured via a human-in-the-loop approach, refined using large language models (LLMs). The MHCLE algorithm performed multi-label classification of 13 SDOMH variables and was evaluated against baseline models, including RoBERTa, Bio_ClinicalBERT, ClinicalBERT, and ClinicalBigBird.

**Results:**

The MHCLE model achieved superior performance with 96.29% accuracy and a 95.41% F1score, surpassing baseline models. Training-testing policies P1, P2, and P3 yielded accuracies of 98.49%, 90.10%, and 89.04%, respectively, highlighting the importance of human intervention in refining LLM annotations.

**Discussion and Conclusion:**

Integrating the MHCLE model with the HLLIA framework offers an effective approach for predicting SDOMH factors from clinical notes, advancing NLP in OUD care. It highlights the importance of human oversight and sets a benchmark for future research.

## Introduction

Social determinants of mental health (SDOMHs) include socioeconomic status, education, community environment, employment, social support, and healthcare access.^1^ Linked to social inequalities, these factors increase mental health risks.^2^ Individuals in adverse social conditions are more susceptible to mental health issues due to structural elements perpetuating cycles of disadvantage and poor health across generations.^3^ Research shows that medical disability, acute pain, and mental disturbances are associated with opioid use disorder (OUD), leading to self-medication and addiction. Understanding these interconnected SDOMHs on OUD is essential for advancing new strategies to improve patient care.^4^

Opioid addiction is influenced by cultural, societal, and individual factors. Those with lower household incomes are more likely to use prescription opioids.^5^ Socioeconomic, psychological, and cultural elements contribute to addiction, highlighting mental health’s importance. Depression and other mental health issues are closely linked to long-term opioid use, both as cause and effect, increasing dependence risk. Treating opioid addiction requires nuanced approaches addressing psychological and SDOMH factors. The relationship between chronic pain, mood disorders, and opioid use necessitates collaborative, multidimensional treatment strategies considering the patient’s mental and social context.^5^^,^^6^

Since the late 90s, the US opioid epidemic has caused over 645 000 deaths by 2021 and significant economic costs.^7^^,^^8^ Clinicians struggle to address SDOMH factors due to limited electronic health record (EHR) recording and a lack of comprehensive understanding, revealing a gap in integrating these factors into research and patient support.^9^ Unstructured data contain about 90 times more SDOH information than structured data.^10^ NLP is crucial for extracting SDOMH from clinical texts but requires extensive expert annotation.^9^ Although LLMs like ChatGPT show strong zero-shot annotation abilities, their alternative use is underexplored.^5^ The costly, error-prone annotation process led to the development of LLM-augmented expert annotation for efficiently annotating SDOMH from patient notes.

Although common OUD risk factors have been identified from structured EHR data,^7^ unstructured EHR data remain underexplored for SDOMH factors linked to OUD. Our nested case-control study used both structured data (eg, ICD codes) and unstructured clinical notes processed by our NLP system from a vast EHR database. This enabled us to investigate the relationship between 13 SDOMHs factors (Table 1) and OUD risk. Our advanced NLP tools extract SDOMHs data from unstructured clinical text, filling a research gap and offering valuable insights for OUD prevention and treatment.^11^ Data annotation is essential but costly for large machine learning datasets. Transfer and active learning reduce labeling costs; crowdsourcing offers scalable, economical annotation. LLMs underpin NLP tasks like sentiment analysis, document categorization, and summarization,^12^ enhancing recommendation systems via fine-tuning and prompt tuning. Open-source LLMs (eg, HugginChat, FLAN) are popular for affordability, reproducibility, and data privacy.^13^ LLMs serve stakeholders, providing accurate answers, especially in medical contexts.^14^

**Table 1.** Overview of our proposed Framework, HLLIA (Human-in-the-Loop-LLM Interaction for Annotation), MIMIC-IV (Medical Information Mart for Intensive Care -IV), and MHCLE (Multilevel Hierarchy Clinical-Longformer Embeddings).

NLP-extracted parameter	Description	Prototype tokens or keywords
Socially detached (SD)	Recognizing the isolation experienced by individuals, the deficit of social engagement or support from relatives, and their matrimonial or partnership conditions. Identifying the paucity of social ties and the resultant strain on family networks	Alone, unaccompanied, reclusive, estranged, lonesome, desolate
Health care handover (HCH)	Shifts in healthcare management, including medication changes or healthcare personnel transitions; updates in patient status involving discharge procedures or patient transfer protocols.	Transfer, handover, treatment change, shift, care passage, service movement
Obstacles to medical care (OMC)	Barriers in effective communication, especially impacting individuals with cognitive impairments; transportation issues that limit access to medical services, and the lack of relational trust or connectivity with healthcare providers.	Accessibility hurdles, medical service barriers, entry obstacles, healthcare blockades, appointment difficulties, resource limitations
Financial uncertainty (FU)	Facing monetary hurdles, experiencing barriers to stable employment, and enduring underprivileged economic states.	Financial insecurity, economic instability, monetary struggle, fiscal hardship, employment uncertainty, economic adversity
Residential instability (RI)	Dealing with residential instability, grappling with concerns over stable shelter, and uncertainties surrounding living conditions.	Homelessness, eviction, housing instability, shelterlessness, residential instability, lodging insecurity
Nutritional shortage (NS)	Insufficient access to balanced and healthy food options, dependency on community support and social welfare for meeting basic dietary needs, and the necessity of utilizing emergency food services.	Hunger, malnutrition, food deficit, nutritional lack, famine, undernourishment
Violence (V)	The existence or potential for violent encounters, experiencing or fearing severe mistreatment or aggression, the impact of discriminatory practices on personal security, and the psychological repercussions of violent thoughts or environments.	Violence, cruelty, viciousness, ferocity, harshness, abuse
Judicial obstacles (JO)	The complexities of navigating legal systems, the repercussions of legal sanctions, the experience of being subject to judicial constraints, and the difficulties arising from legal disputes or criminal accusations.	Legal hurdles, prosecution, legal entanglements, court challenges, legal disputes, juridical complications
Substance misuse (SMI)	The complications associated with excessive or illicit consumption of substances, the medical and social challenges of addiction, and the risks and consequences of acute intoxication leading to overdose.	Substance misuse, addiction, drug dependency, intoxication, overdose, chemical abuse
Mental disturbance symptoms (MDS)	Experiences of profound emotional distress, the social withdrawal associated with mental health issues, barriers to cognitive and emotional regulation, and the patterns that may lead to seeking psychiatric or psychological intervention.	Emotional distress, mental disorder, behavioral symptoms, psychological distress, mental disruption, affective disorder
Acute pain (AP)	Intense and severe physical suffering that may require clinical attention and management, often indicative of a significant underlying health issue.	Torment, severe discomfort, throbbing, sharp pain, intense suffering, piercing agony
Medical disability (MD)	The reliance on support due to medical disabilities, the impact of disabilities on one’s quality of life and autonomy, and the role of medical aids in providing functional assistance.	Functional limitation, physical disability, cognitive impairment, sensory limitation, health disability, medical limitation
Suicide mortality (SM)	Acts or considerations towards ending one’s own life, including the mental states that precede such actions and the tragic outcomes of these considerations.	Self-harm, suicidal ideation, self-inflicted mortality, life-ending act, deliberate self-harm, existential despair

LLMs are underexplored for explainable text annotation due to transparency issues affecting trust. Despite interest in their reasoning abilities, interpretability requires further investigation.^15^ The “explain-then-annotate” LLM approach improves data labeling beyond crowdsourcing, enhancing precision and reliability^16^ but lacks methods to identify successful queries, raising concerns. This study introduces a design strategy prompt using trial-and-error and statistical metrics. An expert-enhanced definition for OUD patients’ Mental Health Datasets improves LLM annotations’ interpretability for expert verification, enhancing multi-class, multi-label classification of SDOMH factors and understanding patient mental health complexities. Generative AI like ChatGPT holds healthcare potential^17^ but faces biases, interpretability issues, and high computational demands.^15^ LLMs inherit data biases,^18^ struggle with reasoning and rare words,^19^ and risk spreading disinformation.^20^ Issues like hallucinations and flawed reasoning demand ethical, responsible development.^11^

**Figure 1.**
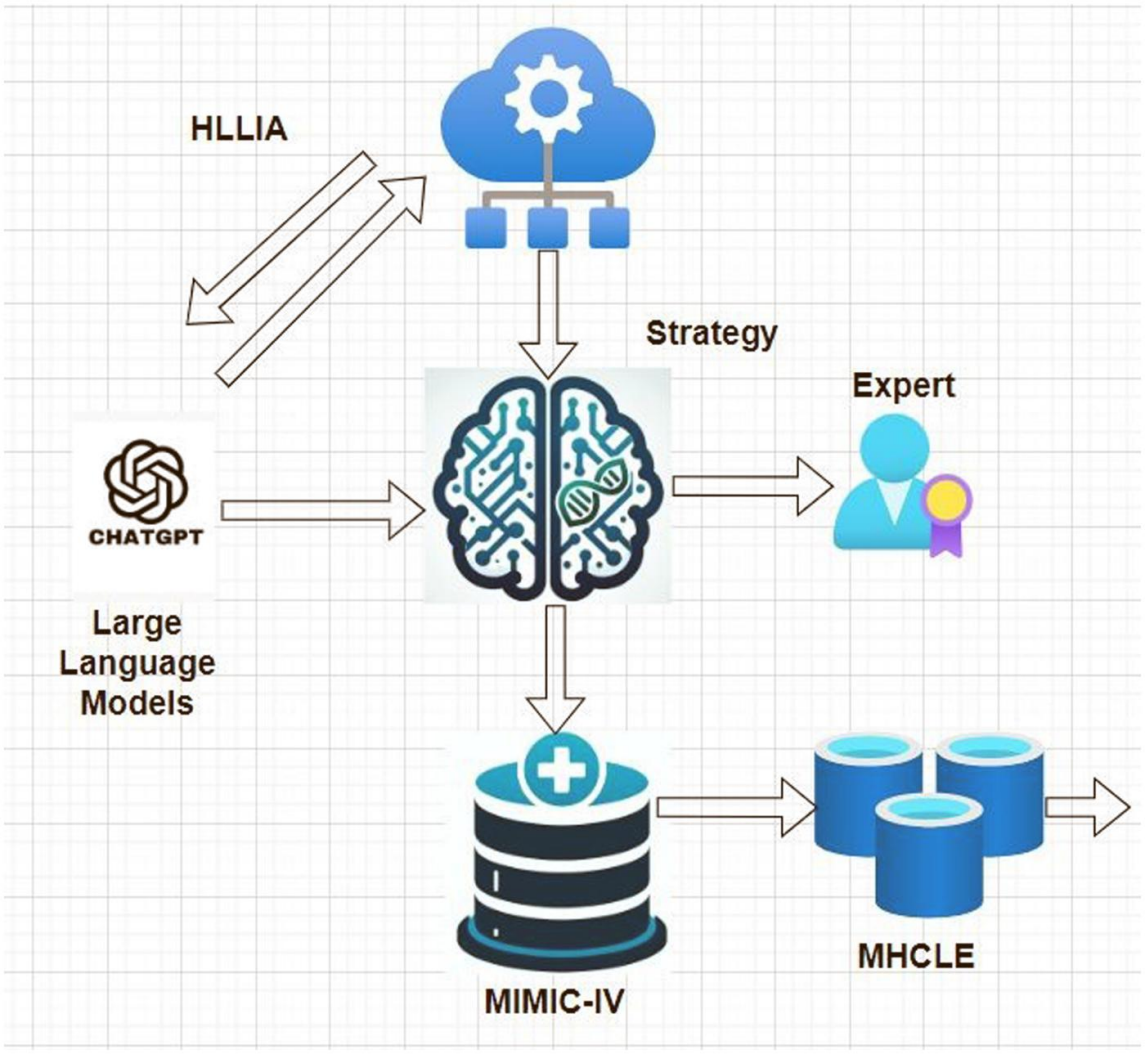
Overview of our proposed framework, HLLIA (Human-in-the-Loop-LLM Interaction for Annotation), MIMIC-IV (Medical Information Mart for Intensive Care -IV), and MHCLE (Multilevel Hierarchy Clinical-Longformer Embeddings).

We proposed an efficient Human-in-the-Loop-LLM Interaction for Annotation (HLLIA) framework to generate an appropriate strategy for LLM-augmented expert annotation of SDOMHs from patients’ clinical notes (MIMIC-IV) (Figure 1).The HLLIA framework refines LLM-generated annotations through expert oversight, ensuring accuracy, and consistency. Additionally, we proposed a Multilevel Hierarchical Clinical-Longformer Embeddings (MHCLE) model, which is used to classify and evaluate the prediction of SDOMHs based on these refined annotations. Our study explores different training and testing strategies, referred to as policies, to evaluate the robustness of the proposed models. These policies compare performance under varying conditions, such as testing on human-annotated versus LLM-generated datasets, to assess model adaptability and accuracy.

## Materials and methods

### Data collection and processing

Despite digital information systems, healthcare providers still use free-text notes for communication and care plans. Discharge summaries—long narratives describing patients’ hospital courses and instructions—are crucial.^9^ Our study uses the MIMIC-IV-Note dataset from PhysioNet, containing 331 794 deidentified discharge summaries from 145 915 patients at BIDMC in Boston, MA, USA.^20^^,^^21^ We focused on the first 2636 rows of “discharge.csv” to balance analysis depth and computational efficiency. Using Python’s pandas and re libraries, we sanitized and standardized the text by removing patient identifiers, unnecessary punctuation, serialization artifacts, and redundant information. We corrected common abbreviations and standardized terms. Advanced NLP techniques via Natural Language Toolkit (NLTK) including tokenization, stop words removal, lemmatization, and handling domain-specific abbreviations—further refined the dataset. This enhanced text quality, producing a curated dataset optimized for analyzing OUD and its management through targeted cleaning and standardization.

We recruited 4 experienced academic mental health researchers, trained them on target SDOMH factors, and had them annotate data from 1495 patients’ (referenced as Subject_id) discharge summaries, totaling 2636 entries.

### Demographics and mental health comorbidities

**Figure 2.**
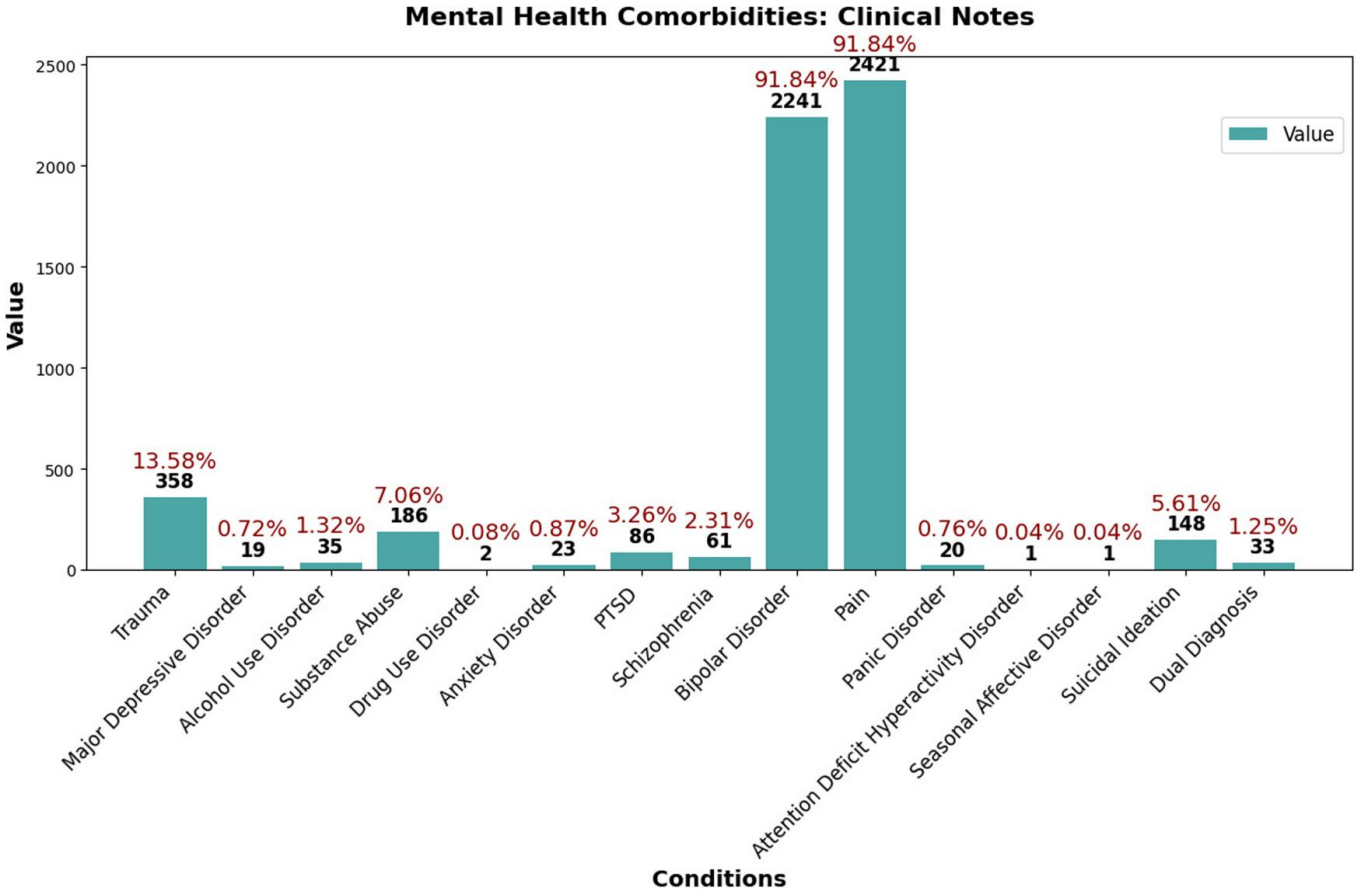
Mental health comorbidities: clinical notes.

We conducted basic analytics to understand demographics and patients’ mental health. Noticing multiple discharge summaries per patient (same subject_id), we removed duplicate subject_ids, resulting in 1426 records (Table 2). We explored mental health comorbidities in clinical notes, identifying records indicating comorbidities using NLP-extracted variables. Comorbidity counts were calculated based on the original 2636 records (Figure 2).

**Table 2.** Basic demographic information of participants.

Characteristics	Value	Percentage (%)
**Sex**		
Female	658	46.14
Male	768	53.86
**Age**		
18-29	214	15.01
30-39	197	13.81
40-49	237	16.62
50-59	248	17.39
60-69	213	14.94
70-79	153	10.73
80-100	164	11.50
**Race**		
American Indian/Alaska Native	5	0.35
Asian	26	1.82
Black/African American	183	12.83
Hispanic/Latino	87	6.10
Other	57	4.00
White	976	68.44
Native Hawaiian or Other Pacific Islander	3	0.21
Portuguese	6	0.42
Multiple race/ethnicity	2	0.14
Unable to obtain	12	0.84
Patient declined to answer	4	0.28
Unknown	65	4.56
**Marital status**		
Married	466	32.68
Single	654	45.86
Divorced	122	8.56
Widowed	118	8.27
Unknown	66	4.63

### SDOMH: opioid use disorder patients

Social determinants of health (SDoH)—socioeconomic status, education, social support are crucial for mental and physical outcomes, linking social inequality to health risks. Halbert et al. found longer term opioid use among US adults with mood disorders.^22^ Despite their importance, SDoH data are often buried in unstructured EHR notes, making NLP vital for data extraction.^11^ Research highlights the need for holistic treatments considering both clinical and social factors. Experts emphasize systemic barriers like racism and classism impact opioid addiction.^6^ Our team identified 13 SDOMH factors crucial for understanding overall well-being and struggles.

### Human-in-the-loop-LLM interaction for annotating gold labels

#### LLMs for annotation

ChatGPT (gpt-3.5-turbo) excels in identifying genres in low-resource languages, while GPT-4 effectively annotates and explains complex texts, aiding interpretative studies.^23^ Our study leveraged both for data annotations. LLMs are crucial in NLP, healthcare, and education, aiding decision-making and question-answering.^15^^,^^17^ As a promising alternative for text annotation, LLMs challenge traditional methods through HLLIA, a novel approach enhancing annotation via their interpretive capabilities.^16^^,^^17^

To reduce human annotators’ workload, we present a methodology named HLLIA. The annotation process had 2 stages: initially, expert annotators labeled a limited dataset without LLMs. Then, using guidelines, they employed LLMs for effective prompt strategies (Figure 6) and statistical quality evaluation. The most effective prompt, determined by the highest partial correlation coefficient, was used to annotate the rest of the dataset. Guidelines included clear prompt objectives, iterative testing, leveraging context, and avoiding ambiguity. Ensuring correlations among SDOMH variables enhances understanding of their complex relationships, improving dataset reliability for analysis.

**Algorithm 1**: Human-in-the-Loop Interaction for Annotation (HLLIA)

**Input:** Determinants of Mental Health for Opioid, Minimal Labeled Dataset ($D_{l}$), Extensive Unlabeled Dataset ($D_{u}$)

**Output:** Annotated Dataset $D_{u}$

is_processing← True;

**while**  is_processing  **do** 

  1. *Select_Strategy* ← Chosen annotation strategy;

  2. *Annotations* ← Extracted annotations and interpretations;

  3. Compute Partial Correlation coefficient (*r*) for SDMHs;

  4. **if**  r>Threshold(T)  **then** 

    is_processing← False;

   **End** 

**End** 

Annotate $D_{u}$ using Strategy-with-augmentation;

**return** Annotated $D_{u}$

#### Enhancing annotation accuracy: HLLIA vs traditional methods

Manual annotation methods are inconsistent, inefficient, and error-prone, especially with large datasets like clinical notes. The HLLIA framework improves these methods by incorporating LLMs for preliminary annotations, followed by expert refinement. HLLIA uses an iterative process where annotations are statistically evaluated using partial correlation coefficients, ensuring high-quality labeling at each step. Only annotations meeting a specific correlation threshold are retained, minimizing errors and enhancing label reliability. This hybrid approach accelerates the annotation process and results in annotations significantly more accurate and consistent than traditional manual methods.

#### Augmenting annotation capabilities with the HLLIA algorithm in LLM

We recruited 4 mental health experts and input our expert-developed SDOMH set for OUD patients based on clinical notes, a minimal labeled dataset (Dl), and an extensive unlabeled dataset (Du) into HLLIA Algorithm 1. Experts were trained on LLM-based query strategies to classify text by multiple SDOMHs and determine their presence. The goal was to achieve both classification and rationale for associating or excluding specific SDOMHs. After LLM annotation, we labeled Dl and computed the partial correlation coefficient, r, for detecting any SDOMH while controlling for the other 13 SDOMH constant.^24^ If r surpassed the threshold Th (set at 0.8),^24^ the query cycle terminated; otherwise, it restarted. Once the desired r was achieved, we applied the strategy to the entire unlabeled dataset, Du, to obtain labeled Du, which was then reviewed to eliminate annotation biases. After 15 iterations, we identified an optimal strategy with r exceeding 0.8 for detecting any SDOMH variables, considering the 13 SDOMH as controlled variables.

#### Leveraging HLLIA iteratively to annotate SDOMHs

The primary objective of the HLLIA system is to streamline text annotation through large language model (LLM) augmented explanations. Initially, an expert manually annotated 200 texts for SDOMHs from OUD clinical notes. Subsequently, 4 mental health experts were trained by our team on using ChatGPT^23^ within HLLIA. They were guided by predefined strategies to identify effective approaches, aiming for a partial correlation coefficient (r) greater than 0.8 (Section 5). Experts also considered the explainability of LLM-generated annotations. Once the optimal strategy was identified, annotators reviewed and refined the LLM-generated annotations, ensuring accuracy and preserving both LLM and expert-augmented annotations.

### Multilevel Hierarchical Clinical-Longformer Embeddings (MHCLE) classification algorithm

#### Notations and preliminaries

We denote the input clinical notes text as X. The set of labels for hierarchical levels H_1_, H_2_, and H_3_ are denoted as Y_1_, Y_2_, and Y_3_, respectively. For H_1_ and H_2_, the task is binary classification, where Y_1_, Y_2_= {0, 1}, indicating the absence or presence of OUD in H_1_ and social determinants in H_2_. For H_3_, which is a multilabel classification task, Y_3_ can take multiple labels indicating the presence of multiple SDOMH variables that are applicable from a predefined set.

### Model architecture

We proposed a Multilevel Hierarchical Clinical Text Classification framework using the Clinical-Longformer model. The architecture is designed to leverage the Clinical-Longformer’s ability to process lengthy clinical narratives and extract meaningful embeddings. (i) Hierarchy 1: Binary Classification for H_1_: The initial task is to classify clinical text X into 2 categories: indicating whether or not there is OUD. The Clinical-Longformer in the embedding layer transforms X into a high-dimensional vector space. The dense layer, with 64 hidden units and a Rectified Linear Unit (ReLU) activation function, refines the embeddings for this classification level. The output layer, with a single neuron and a sigmoid activation function, computes the probability that X belongs to the presence or absence of OUD. (ii) Hierarchy 2: Binary Classification for H_2_: The model then proceeds to Task 2 for binary classification within the broad category determined previously, focusing on mental health determinants. The dense layer, using refined embeddings with 32 hidden units and ReLU activation, captures necessary nuances. The output layer uses a sigmoid activation function to determine the probability that X falls into 1 of the 2 categories of social determinants in H_2_, indicating its presence or absence. (iii) Hierarchy 3: Multilabel Classification for H_3_: This task is activated if the clinical text X is recognized to have the presence of Social determinants from H_2_, requiring further classification to identify which of the 13 SDOMH variables are applicable. In the multi-label dense layers, each label in H_3_ has a corresponding dense layer, starting with 128 hidden units and ReLU activation to handle non-linearity. The output layer uses individual sigmoid functions to predict the probability of each SDOMH label’s presence. Our hierarchical framework efficiently processes and categorizes clinical texts into multi-level labels, enabling deep semantic understanding and nuanced classification of clinical narratives.

### Optimizing model: loss functions and training techniques

The final loss (L_Total_) function is a weighted sum of the Binary Cross-Entropy (BCE) loss used for all hierarchical levels. Let α be the weighted coefficient for hierarchy 1, β be the weighted coefficient for hierarchy 2, and $\gamma_{1},\gamma_{2},\gamma_{3},\gamma_{4},\ldots ,\gamma_{13}$ be the weighted coefficients for hierarchy 3, which correspond to the 13 SDoMHs labels. The Multilevel hierarchical Clinical-Longformer embedding loss function can be defined as $L_{\text{Total}}$, shown in Equation 1:


(1) $$\begin{array}{c} L_{\text{Total}}=\alpha (-\frac{1}{N}\sum_{i=1}^{N}[y_{1,i}\;\text{log}({\hat{y}}_{1,i})+\\ +(1-y_{1,i})\;\text{log}(1-{\hat{y}}_{1,i})])\\ +\beta (-\frac{1}{N}\sum_{i=1}^{N}[y_{2,i}\;\text{log}({\hat{y}}_{2,i})+\\ +(1-y_{2,i})\;\text{log}(1-{\hat{y}}_{2,i})])\\ +\gamma (-\frac{1}{N}\sum_{i=1}^{N}\sum_{j=1}^{13}[y_{3,ij}\;\text{log}({\hat{y}}_{3,ij})+\\ +(1-y_{3,ij})\;\text{log}(1-{\hat{y}}_{3,ij})]) \end{array}$$

For binary classification tasks H_1_ ($y_{1,i}$ and ${\hat{y}}_{1,i}$) and H_2_ ($y_{2,i}$ and ${\hat{y}}_{2,i}$): *y* and $\hat{y}$ represent the actual labels and predicted probabilities, respectively, for the *i*-th sample. For multi-label classification tasks H_3_ ($y_{3,ij}$ and ${\hat{y}}_{3,ij}$): *y* and $\hat{y}$ indicate the actual labels and predicted probabilities for each of the 13 labels, respectively, for the *i*-th sample and *j*-th label.

The hierarchical model is trained by iteratively minimizing the total loss using backpropagation and an optimizer like Adaptive Moment Estimation with Decoupled Weight Decay (AdamW). This updates the model’s parameters to maximize performance in H_1_, H_2_, and H_3_ classification. The model’s architecture, including the Clinical-Longformer for text encoding, is designed to handle the complexity of hierarchical tasks, extracting and learning nuanced features for classification. By reducing the total loss iteratively, the training procedure fine-tunes the model to improve prediction accuracy. Note: The specific value of the weight coefficients α and β has been determined based on the relative importance of each task (0.8>), and $\gamma_{j}$ has been determined based on hyperparameter tuning ranging from 0 to 0.5.

We built a custom accuracy metric for H_3_ given its multi-label nature. The custom accuracy calculation computes the proportion of correctly predicted labels to the total number of labels per sample, then averages these proportions across all samples. Custom precision, recall, and F1 metrics are calculated per sample based on true positives, false positives, and false negatives (missed labels). Averages of these per-sample metrics are reported as the final precision, recall, and F1 for H_3_. H_3_ predictions are also binarized using a threshold of (0.5). To further elaborate on the custom evaluation metrics for H_3_, for custom precision, we first compute the precision for each sample by dividing the number of true positive predictions by the sum of true positives and false positives for that sample. This calculation ensures that precision reflects the model’s ability to correctly identify relevant labels among the predicted ones. Custom recall is computed for each sample by dividing the number of true positives by the sum of true positives and false negatives, capturing the model’s capacity to find all relevant labels. The F1 score, which balances precision and recall, is calculated per sample using the harmonic mean of precision and recall. We then average these per-sample precision, recall, and F1 scores across all samples to obtain the final metrics. This approach provides a detailed understanding of the model’s performance on a per-sample basis, ensuring that the evaluation is comprehensive and accurately reflects the multi-label classification scenario.

### Baseline models and experimental evaluation

#### Datasets details

**Figure 3.**
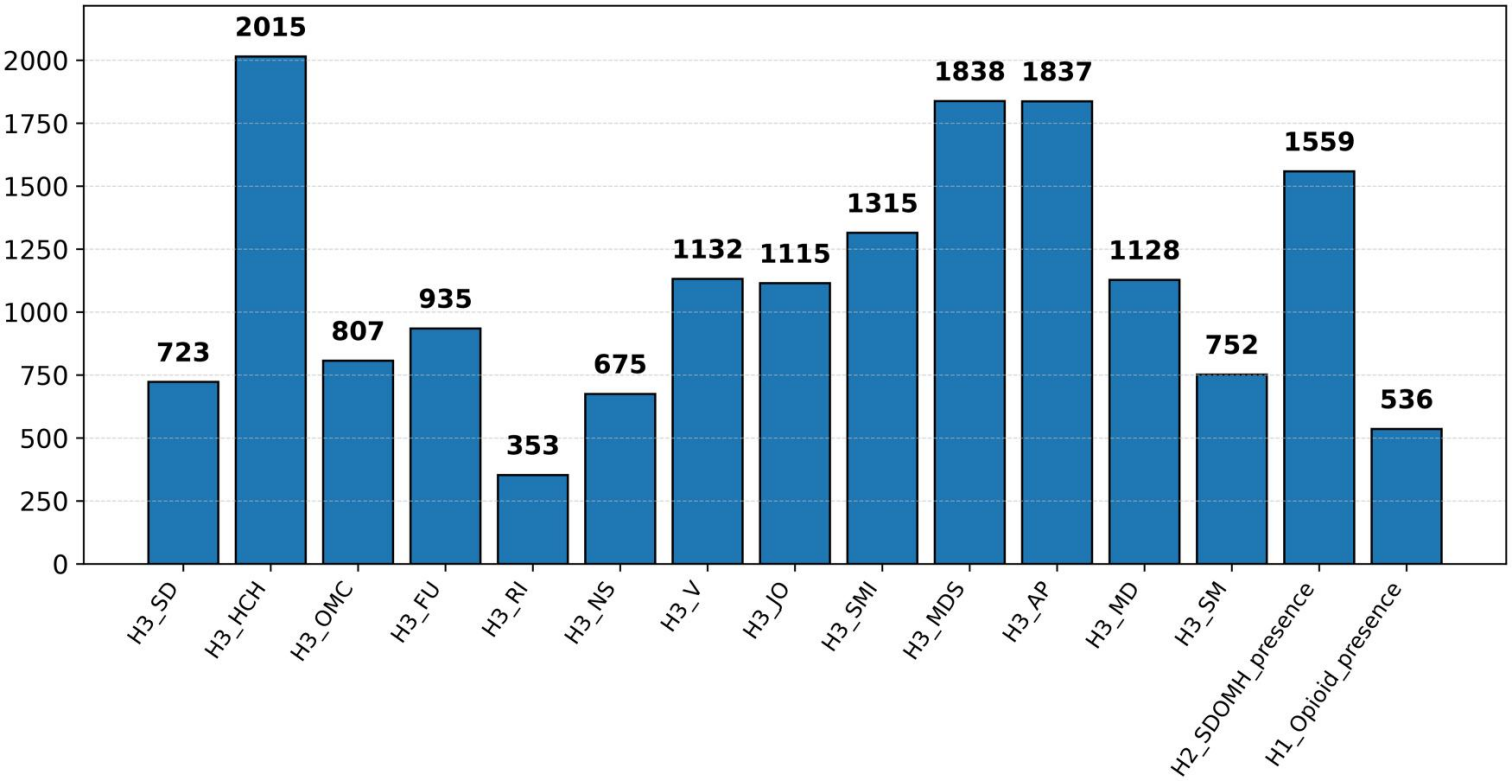
HLLIA annotated class distribution for 13 different SDOMHs and absence/presence of any of the 13 SDOMHs. Here, “SD,” “HCH,” “OMC,” “FU,” “RI,” “NS,” “V,” “JO,” “SM,” “MDS,” “AP,” “MD,” and “SM” refer to Socially Detached, Health Care Handover, Obstacles to Medical Care, Financial Uncertainty, Residential Instability, Nutritional Shortage, Violence, Judicial Obstacles, Substance Misuse, Mental Disturbance Symptoms, Acute Pain, Medical Disability, and Suicide Mortality, respectively.

In our study, we have generated 3 datasets. D1: 212 small amount of Clinical notes texts along with human annotated labels of SDOMH variables. D2: 2636 Clinical notes texts with HLLIA augmented expert annotation and solely LLM annotation of SDOMH variables. D3: 2636 LLM (ChatGPT) generated texts that have already HLLIA augmented expert annotation as well as solely LLM annotation of SDOMH variables. Figure 3 shows HLLIA based annotated class distributions in the D2 dataset.

#### Proposed MHCLE model performance for SDOMHs prediction

We have implemented a MHCLE algorithm using python based PyTorch platform. Our MHCLE model training-testing policies are as follows:

#### Training-testing policies

- **(P1)**: Trained on D2 dataset (original Clinical notes texts) taking HLLIA annotations as labels and tested on D2 dataset with HLLIA annotations as labels (70%/30% split).
- **(P2)**: Trained on D2 dataset taking HLLIA annotations as labels (100%) and tested on D1 dataset (human annotated labels).
- **(P3)**: Trained on D2 dataset taking HLLIA annotations as labels (100%) and tested on D3 dataset (ChatGPT generated Clinical notes equivalent texts).

#### Baseline algorithms

Apart from our proposed MHCLE model, we implemented algorithms for multi-label problems using Hugging Face and PyTorch libraries. Our problem type was “multilabel_classification,” using a sigmoid activation function in the neural network’s classification layer, with decisions based on a threshold exceeding 0.5. We leveraged various baseline models, including RoBERTa, ClinicalBERT, Bio_ClinicalBERT, Clinical-BigBird, and Clinical-Longformer, for our analysis.^25^ Clinical-Longformer, a domain-enriched language model, is pre-trained on extensive clinical corpora and extends the input sequence length from 512 to 4096 tokens using a sparse attention mechanism to reduce memory usage. This model captures long-term dependencies for optimal clinical NLP results and enhances short sequence tasks by providing richer contextualization of clinical concepts.^25^

## Results

### Classification performance

**Figure 4.**
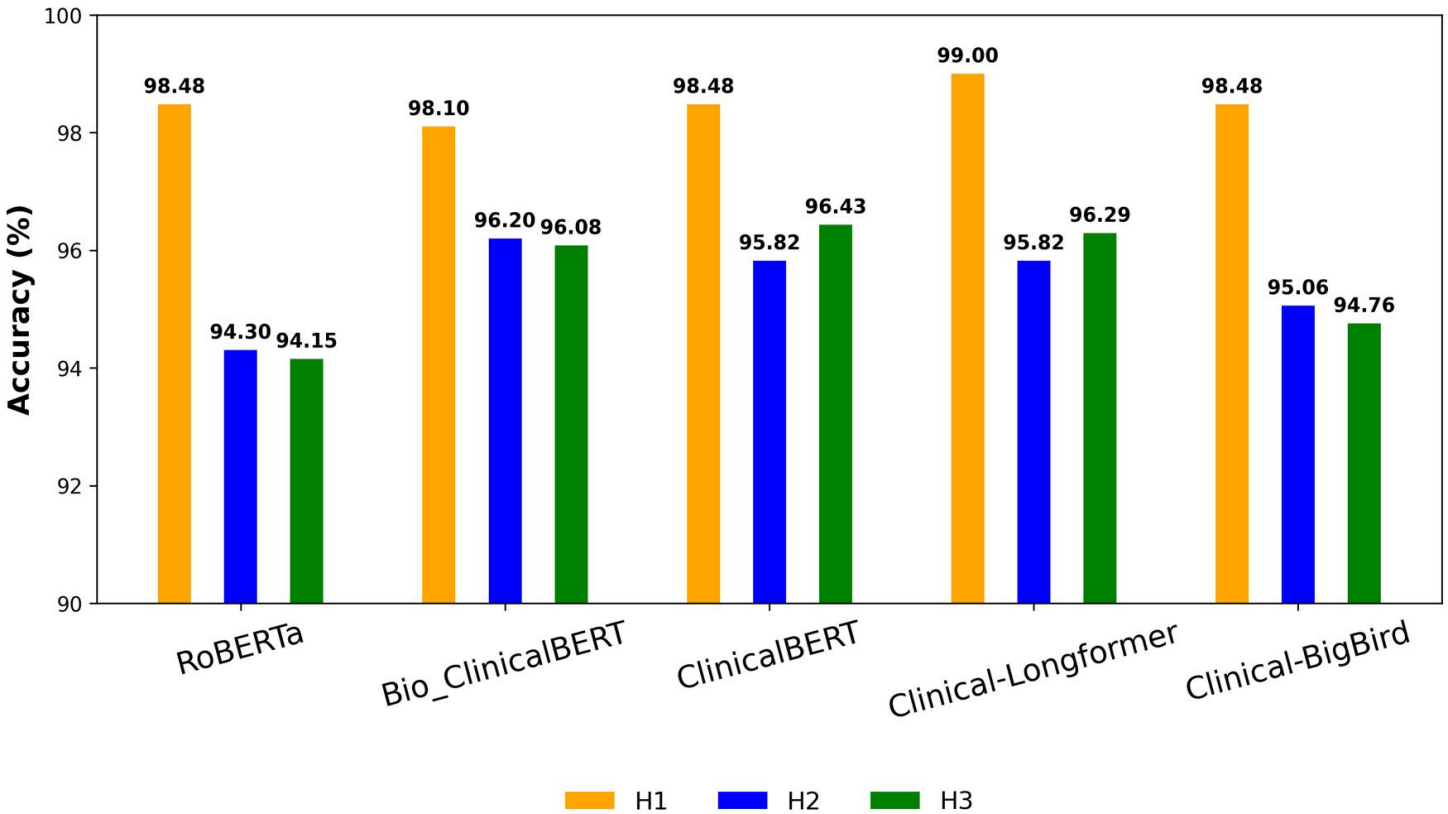
(P1) Performance comparisons of multilevel version of baseline algorithms vs proposed MHCLE with different pre-trained embedding as backbone. Here, the model was trained on D2 Dataset and HLLIA annotated labels.

Table 3 presents the accuracy, F1-score, precision, and recall for training results performance comparisons among different models: RoBERTa, Bio_ClinicalBERT, ClinicalBERT, Clinical-Longformer, and Clinical-BigBird, based on Multilevel classifications. After comparing the Multilevel versions of baseline algorithms with our proposed MHCLE framework using different pre-trained embeddings as the backbone, we observed that the MHCLE(Clinical-Longformer) model significantly outperforms its corresponding baseline multilevel algorithms. Among all the models, Clinical-Longformer embeddings provide the highest accuracy (see Figure 4).

**Table 3.** (P1) Details performance of Multilevel version of baseline algorithms vs proposed MHCLE with different pre-trained embedding as backbone.

Model	Metric	H_1_	H_2_	H_3_
RoBERTa	Acc	98.48	94.30	94.15
	F1	98.47	94.31	92.62
	Prec	98.48	94.30	94.07
	Recall	98.48	94.30	91.22
Bio_ClinicalBERT	Acc	98.10	96.20	96.08
	F1	98.10	96.21	95.16
	Prec	98.11	96.20	95.78
	Recall	98.10	96.20	94.54
ClinicalBERT	Acc	98.48	95.82	96.43
	F1	98.48	95.82	95.65
	Prec	98.48	95.82	96.30
	Recall	98.48	95.82	95.01
Clinical-Longformer	Acc	99.00	95.82	96.29
	F1	99.00	95.82	95.41
	Prec	99.00	95.82	96.03
	Recall	99.00	95.82	94.81
Clinical-BigBird	Acc	98.48	95.06	94.76
	F1	98.48	95.10	93.60
	Prec	98.47	95.06	93.73
	Recall	98.48	95.06	93.48

Here, the model was trained on D2 Dataset and HLLIA annotated labels.

Table 4 showcases the prediction accuracies of MHCLE across a 3-tier hierarchy focused on OUD, its associated social determinants, and the identification of 13 specific SDOMH variables, evaluated across 3 distinct policies (Policy P1, Policy P2, and Policy P3). Here, P1 (trained and tested on the D2 dataset) provided the highest accuracy, signifying that the HLLIA model captures appropriate patterns. P2 (trained on D2 and tested on D1) showed slightly lower, but still comparable, accuracy to P1, indicating that our proposed HLLIA annotations could predict human-annotated texts with high accuracy. However, for policy P3 (trained on D2 and tested on D3), accuracy decreased significantly, indicating that the HLLIA annotation-based MHCLE model struggles to predict texts generated by ChatGPT.

**Table 4.** Performance of multilevel hierarchy version of proposed MHCLE using different pre-trained embeddings on various datasets and annotation types.

Metric	P1			P2			P3		
	H_1_	H_2_	H_3_	H_1_	H_2_	H_3_	H_1_	H_2_	H_3_
**Acc**	99.00	98.90	98.49	98.59	97.19	90.10	93.96	85.73	89.04
**F1**	99.00	98.90	98.19	98.59	97.19	85.34	93.96	85.73	86.46
**Prec**	99.00	98.90	98.44	98.59	97.19	80.90	93.96	85.73	85.74
**Recall**	99.00	98.90	97.93	98.59	97.19	90.52	93.96	85.73	87.24

Besides accuracy, Table 4 also showcases detailed metrics-precision, recall, and F1-score across different policies (P1, P2, and P3).We compared the multilevel hierarchies of baseline algorithms with our MHCLE framework across the 3 policies. Our analysis evaluated test accuracy, consistency, and robustness. The results show that the MHCLE (Clinical-Longformer) model exceeds benchmarks in accuracy and adaptability, making it the top-performing model for clinical analytics and improving healthcare interventions for OUD and related social factors.

#### Association studies over all dataset

**Figure 5.**
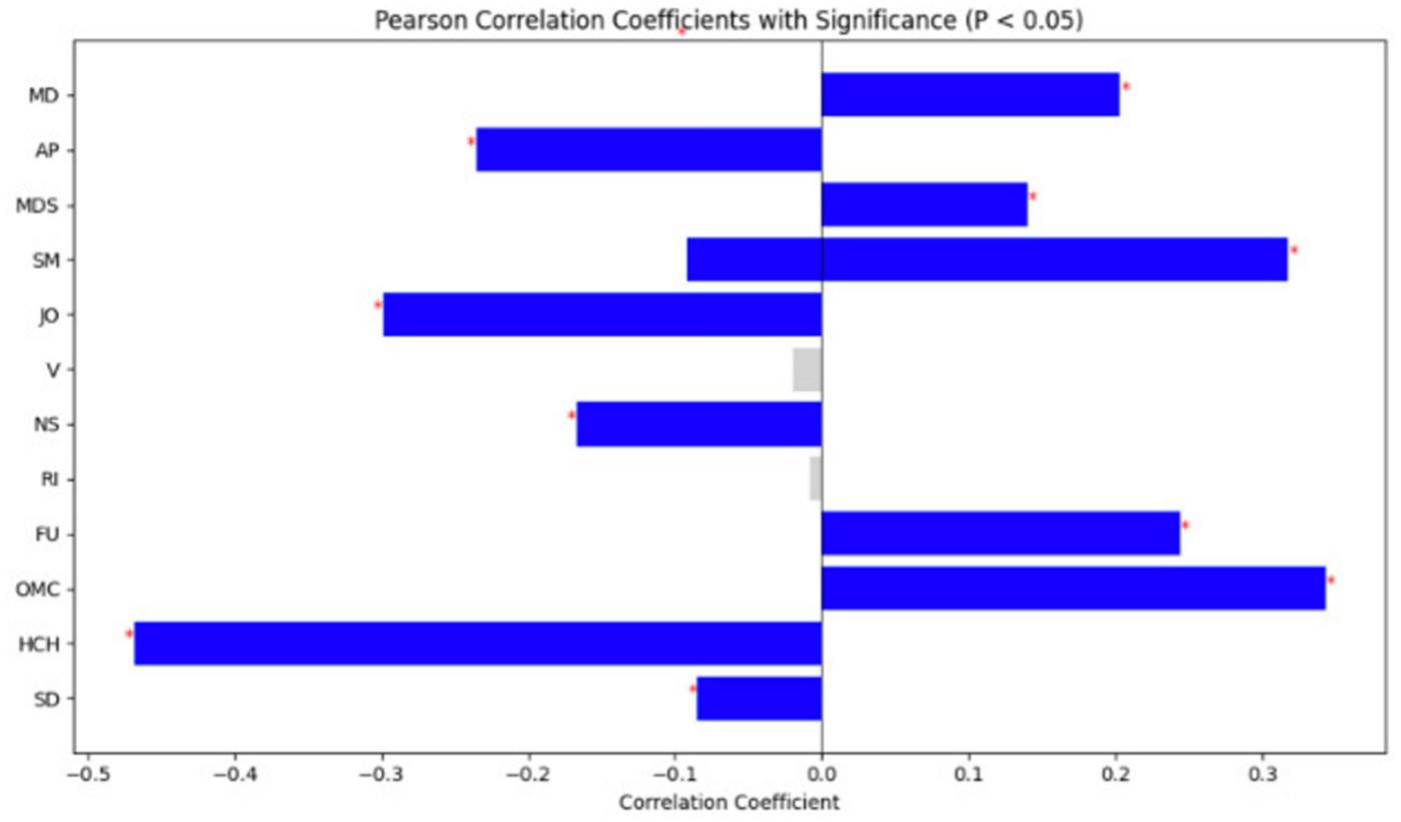
Correlation coefficients of different SDOMHs with significance*.

We ran the final developed model to predict 13 SDOMHs and OUD across the entire 331K dataset. We performed a Pearson correlation coefficient analysis with a significance level of *P* < .05, which is shown in Figure 5. The results indicate that Medical Disability (MD), Acute Pain (AP), Mental Disturbance Symptom (MDS), Substance Misuse (SM), Judicial Obstacles (JO), Nutritional Shortage (NS), Financial Uncertainty (FU), Obstacles to Medical Care (OMC), Health Care Handover (HCH), and Socially Detached (SD) have significant correlations with OUD.

## Discussion

We found that our proposed MHCLE model achieved superior performance with 96.29% accuracy and 95.41% F1-score, surpassing baseline models. We designed Training-Testing policies P1, P2, and P3 for the MHCLE model. The model accuracies for Policies P1, P2, and P3 were 98.49%, 90.10%, and 89.04%, respectively. As shown in Figure 4, P1 achieved the highest accuracy within the MHCLE framework. When trained and tested on the same dataset (D2), the model captured OUD patterns and social determinants with high precision. Despite a slight accuracy drop in P2 (trained on D2, tested on D1), the performance remained robust, demonstrating the HLLIA framework’s effectiveness across different datasets. However, P3 showed a notable performance decline with ChatGPT-generated clinical notes, which underscores challenges in adapting to varied linguistic patterns. These results guide future strategies for improving model resilience against data source variations.

The quality and consistency of annotations played a critical role in the performance of hierarchical classification models, particularly for multi-label tasks like ours. The HLLIA framework’s iterative process ensures continuous refinement of annotations, using partial correlation coefficients to statistically evaluate and optimize label quality. This led to cleaner and more reliable data for model training, as evidenced by the improved performance metrics (accuracy, precision, recall, and F1 score) observed in P1. In contrast, annotations produced without this rigorous refinement process, as seen in P3, resulted in lower performance, underscoring the value of combining LLM-generated annotations with human oversight.

For P1, the combination of LLM and human annotations resulted in the highest accuracy, demonstrating that human correction of LLM-generated labels is crucial. P2’s performance, although slightly lower, remained high, reflecting the quality of human-only annotations. The significant drop in P3’s accuracy reveals that our model struggles with ChatGPT-generated text, likely due to differences in style, structure, or content not aligned with the training data. While Named Entity Recognition (NER) tasks have succeeded with LLM-only labels, our task requires deeper domain-specific understanding, which LLMs alone may not provide. Thus, human oversight remains essential to ensure high-quality annotations and model reliability.

### Impact of HLLIA on model performance

The comparative analysis of P1, P2, and P3 underscores the critical role of the HLLIA framework in enhancing model performance. P1, trained and tested on HLLIA-annotated clinical notes (D2), achieved the highest accuracy due to HLLIA’s iterative process and statistical evaluation. P2, tested on human-annotated data (D1), also performed well, demonstrating that HLLIA annotations generalize effectively. However, P3, tested on ChatGPT-generated notes (D3), showed significantly lower accuracy, highlighting the challenges of applying HLLIA-labeled data to synthetic texts. These findings reinforce the strength of HLLIA’s combined approach in producing reliable, high-quality labels that boost model robustness and accuracy.

### HLLIA experience investigation

The interaction between humans and LLMs offers novel research insights. Our 4 expert annotators highlighted ChatGPT’s exceptional text annotation capabilities, providing multi-faceted annotations and clarifications on demand, which simplifies complex tasks. ChatGPT’s integration ensures uniform output, minimizes human bias, and accelerates text analysis, making it ideal for projects with tight deadlines. Despite some incorrect annotations, ChatGPT’s explanation feature aids quick correction, improving overall accuracy. This approach suggests a paradigm shift towards inclusive, citizen-led science research. Annotators’ proficiency improved through continual interaction with ChatGPT, refining their understanding of text annotation nuances.

**Figure 6.**
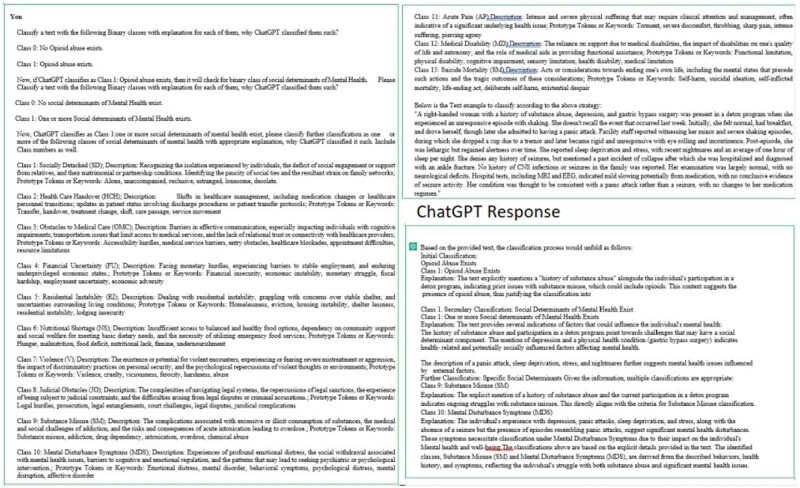
Our proposed framework, HLLIA showcases example of interactions and the resulting text responses generated.

Our study examined ChatGPT’s potential to streamline mental health data annotation. While AI was faster than manual methods, ChatGPT’s labels were sometimes inconsistent. Despite reducing human bias and workload, inconsistencies remained. ChatGPT shows promise for identifying social factors linked to mental health, allowing quicker intervention, but cannot fully replace human involvement. Combining AI and human expertise could lead to faster responses. We defined SDOMH variables from Classes 1-13 and used ChatGPT for annotating labels for a given text (Figure 6).

After evaluating the text with ChatGPT, it was assigned to Class 2: Health Care Handover (HCH) and Class 9: Substance Misuse (SM). However, further interaction revealed no match with predefined categories. Another analysis assigned it to a new category, Class 10: Mental Disturbance Symptoms (MDS). Our findings suggest ChatGPT’s classification accuracy is inconsistent, sometimes misclassifying or failing to classify texts. This limitation highlights ChatGPT’s dependency on learned patterns, hindering its ability to fully understand complex text nuances, leading to potential misclassifications or inaccuracies as seen in our observations.

## Conclusion

This study used LLMs to predict SDOMH factors associated with OUD from clinical notes. We introduced the HLLIA framework and MHCLE classification algorithm to improve annotation and prediction of SDOMH factors on OUD. However, LLMs like ChatGPT can lack human insight, leading to misinterpretations, highlighting the need for human expertise and validation. Future studies should refine LLMs to address authenticity, privacy, cultural sensitivity, and bias issues, ensuring reliable and ethical use in mental health. This study advances mental health diagnostics and sets a benchmark for AI integration in healthcare, specifically in understanding and addressing complex factors influencing OUD.

## Supplementary Material

ooaf142_Supplementary_Data

## Data Availability

The data underlying this article will be accessible from this link: https://doi.org/10.5061/dryad.d51c5b0h7. Also, here is the GitHub repository link for reference: GitHub Repository.

## References

[1] Working Group for Monitoring Action on the Social Determinants of Health. Towards a global monitoring system for implementing the Rio Political Declaration on Social Determinants of Health: developing a core set of indicators for government action on the social determinants of health to improve health equity. Int J Equity Health 2018;17:136 30185200 10.1186/s12939-018-0836-7 PMC6126010

[2] Allen J , Balfour R, Bell R, Marmot M. Social determinants of mental health. Int Rev Psychiatry. 2014;26:392-407. 25137105 10.3109/09540261.2014.928270

[3] Kirkbride JB , Anglin DM, Colman I, et al The social determinants of mental health and disorder: evidence, prevention and recommendations. World Psychiatry. 2024;23:58-90. 38214615 10.1002/wps.21160 PMC10786006

[4] Gnanapragasam SN , Wright LA, Pemberton M, Bhugra D. Outside/inside: social determinants of mental health. Ir J Psychol Med. 2023;40:63-73. 34193324 10.1017/ipm.2021.49

[5] Li M , Shi T, Ziems C, et al 2023. Coannotating: uncertainty-guided work allocation between human and large language models for data annotation. arXiv, preprint arXiv:231015638, preprint: not peer reviewed.

[6] Essien-Aleksi IE , Zhang Y, Koren A, Palacios N, Falcon LM, Tucker KL. Sociocultural factors associated with persistent prescription opioid use (PPOU) among Puerto Rican adults in Massachusetts. PLoS One. 2023;18:e0290104. 37607191 10.1371/journal.pone.0290104 PMC10443880

[7] Mapua AM. The Cost of Culture: Examining a Sociological Model of the Opiod Epidemic in the United States. Carnegie Mellon University; 2018.

[8] Centers for Disease Control and Prevention. Opioid data analysis and resources. September 17, 2024. https://www.cdc.gov/opioids/data/analysis-resources.html

[9] Dorr D , Bejan CA, Pizzimenti C, Singh S, Storer M, Quinones A. Identifying patients with significant problems related to social determinants of health with natural language processing. In: MEDINFO 2019: Health and Wellbeing e-Networks for All. IOS Press; 2019: 1456–1457. 10.3233/SHTI190482 31438179

[10] Mitra A , Pradhan R, Melamed RD, et al Associations between natural language processing–enriched social determinants of health and suicide death among US veterans. JAMA Netw Open. 2023;6:e233079. 36920391 10.1001/jamanetworkopen.2023.3079 PMC10018322

[11] Patra BG , Sharma MM, Vekaria V, et al Extracting social determinants of health from electronic health records using natural language processing: a systematic review. J Am Med Inform Assoc. 2021;28:2716-2727. 34613399 10.1093/jamia/ocab170 PMC8633615

[12] Tillmann C , Trivedi A, Bhattacharjee B. 2024. Efficient models for the detection of hate, abuse and profanity. arXiv, preprint arXiv:240205624, preprint: not peer reviewed.

[13] Alizadeh M , Kubli M, Samei Z, et al 2023. Open-source large language models outperform crowd workers and approach ChatGPT in text-annotation tasks. arXiv, preprint arXiv:230702179, preprint: not peer reviewed.

[14] Chang Y , Wang X, Wang J, et al A survey on evaluation of large language models. ACM Trans Intell Syst Technol. 2024;15:1-45.

[15] Hadi MU , Al Tashi Q, Shah A, et al 2024. Large language models: a comprehensive survey of its applications, challenges, limitations, and future prospects. Authorea Preprints 2023;1:1-26. https://www.researchgate.net/publication/372258530_Large_Language_Models_A_Comprehensive_Survey_of_its_Applications_Challenges_Limitations_and_Future_Prospects.

[16] He X , Lin Z, Gong Y, et al AnnoLLM: making large language models to be better crowdsourced annotators. In: *Proceedings of the 2024 Conference of the North American Chapter of the Association for Computational Linguistics: Human Language Technologies (Volume 6: Industry Track)*, pp. 165-190, Mexico City, Mexico. Association for Computational Linguistics; 2023.

[17] Yu P , Xu H, Hu X, Deng C. Leveraging generative AI and large language models: a comprehensive roadmap for healthcare integration. Healthcare. 2023;11:2776. MDPI 37893850 10.3390/healthcare11202776 PMC10606429

[18] Hacker P , Engel A, Mauer M. Regulating ChatGPT and other large generative AI models. In*:* Proceedings of the 2023 ACM Conference on Fairness, Accountability, and Transparency. ACM. 2023;1112–1123. 10.1145/3593013.3594102 PMC10661580 37990734

[19] Wolf Y , Wies N, Avnery O, Levine Y, Shashua A. 2023. Fundamental limitations of alignment in large language models. arXiv, preprint arXiv:230411082..

[20] Khowaja SA , Khuwaja P, Dev K, Wang W, Nkenyereye L. ChatGPT needs spade (sustainability, privacy, digital divide, and ethics) evaluation: a review. Cognit Comput. 2024;16:2528-2550.

[21] Johnson A , Pollard T, Horng S, et al MIMIC-IV-Note: deidentified free-text clinical notes (latest version). PhysioNet. 2022. 1013026/1cjn-2370

[22] Quinlan J , Alam F, Knox K. Opioid analgesic dependence: where do we go from here? Br J Gen Pract. 2017;67:154-155. 28360045 10.3399/bjgp17X690065 PMC5565835

[23] Open AI. ChatGPT (Mar 14 version) [large language model]. 2023. Accessed March 14, 2023. https://chatgpt.com/.

[24] Zbiciak A , Markiewicz T. A new extraordinary means of appeal in the Polish criminal procedure: the basic principles of a fair trial and a complaint against a cassatory judgment. AJEE. 2023;6:25-42.

[25] Li Y , Wehbe RM, Ahmad FS, Wang H, Luo Y. 2022. Clinical-Longformer and Clinical-BigBird: transformers for long clinical sequences. arXiv, preprint arXiv:220111838.

