# Supplementary figures and images for: Augmenting large language models to predict social determinants of mental health in opioid use disorder using patient clinical notes
Source: JAMIA Open. 2025 Nov 27;8(6):ooaf142. doi: 10.1093/jamiaopen/ooaf142 (PMC12664681; doi:10.1093/jamiaopen/ooaf142)

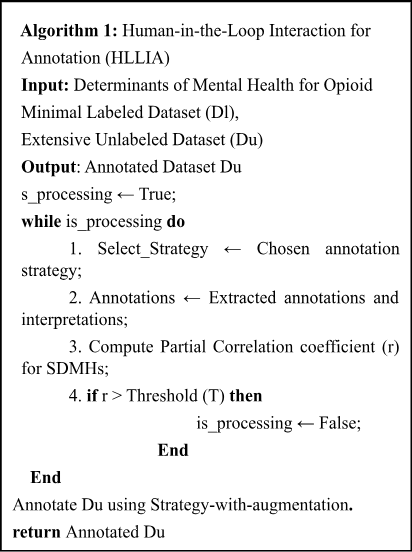

Supplement: ooaf142_Supplementary_Data [file ooaf142_supplementary_data.zip › Algorithm (1).png]

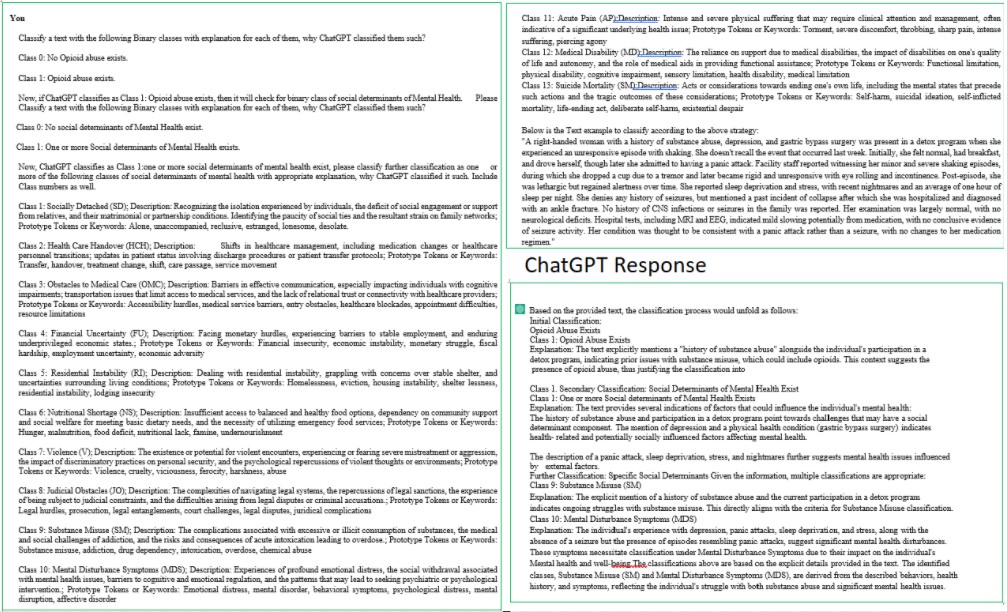

Supplement: ooaf142_Supplementary_Data [file ooaf142_supplementary_data.zip › Chatgpt prompt and response (3).jpg]

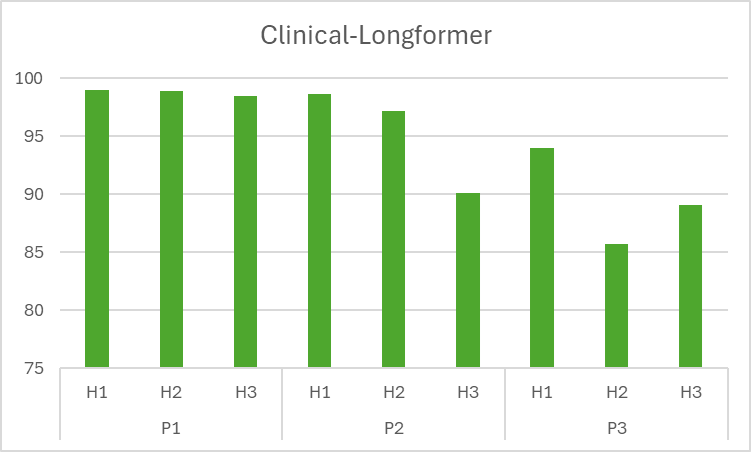

Supplement: ooaf142_Supplementary_Data [file ooaf142_supplementary_data.zip › Clinical Longformer across different policies based on accuracies (2).png]

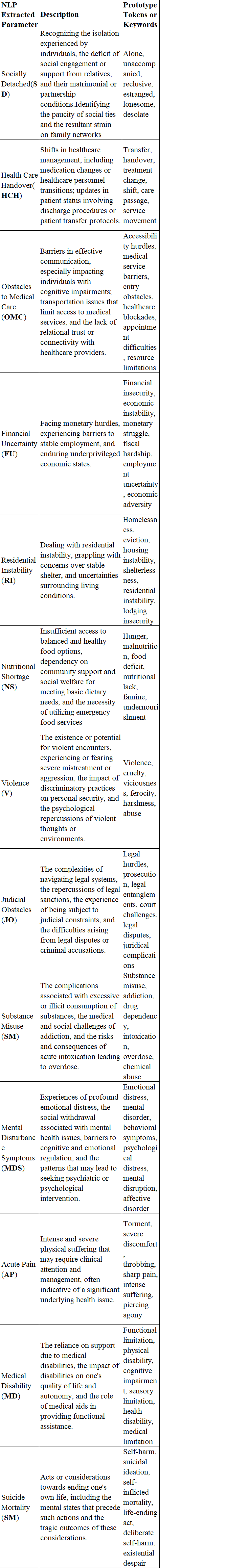

Supplement: ooaf142_Supplementary_Data [file ooaf142_supplementary_data.zip › Determinants table.png]

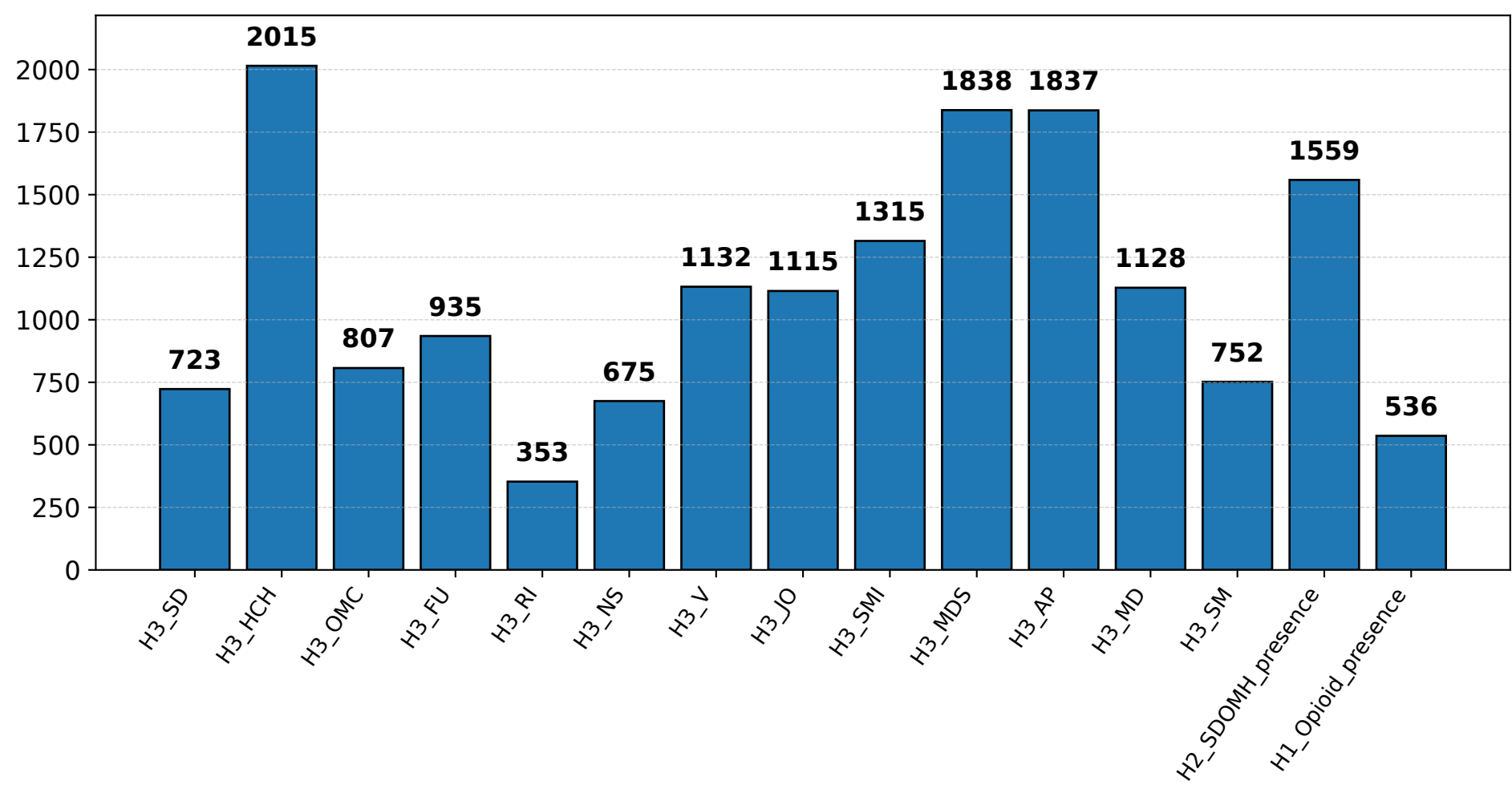

Supplement: ooaf142_Supplementary_Data [file ooaf142_supplementary_data.zip › F_class_distribution.pdf]

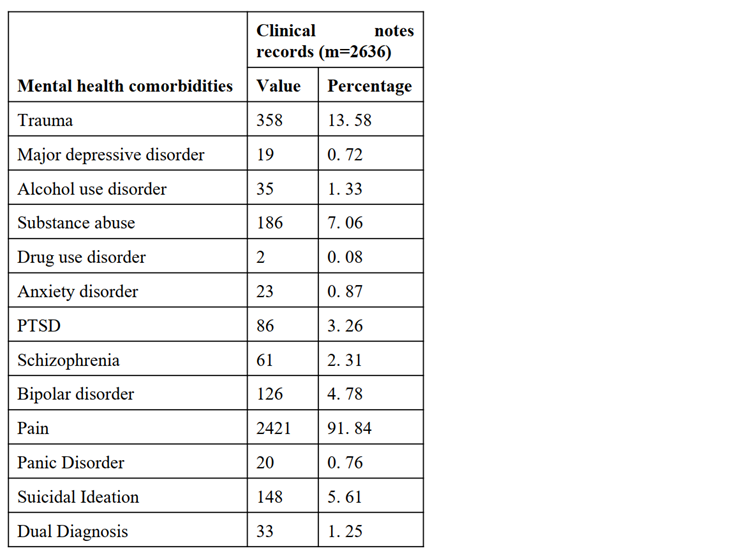

Supplement: ooaf142_Supplementary_Data [file ooaf142_supplementary_data.zip › Mental Health Comorbities (1).png]

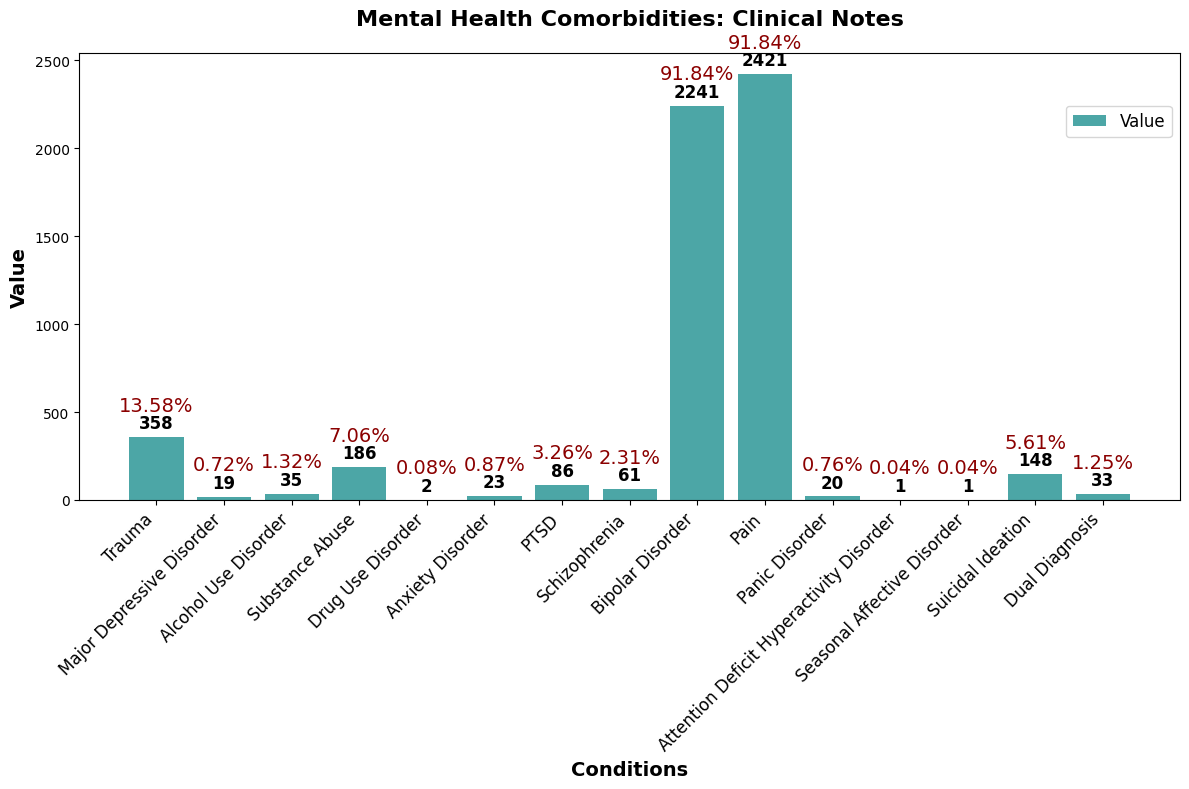

Supplement: ooaf142_Supplementary_Data [file ooaf142_supplementary_data.zip › Mental Health Comorbities_v1.png]

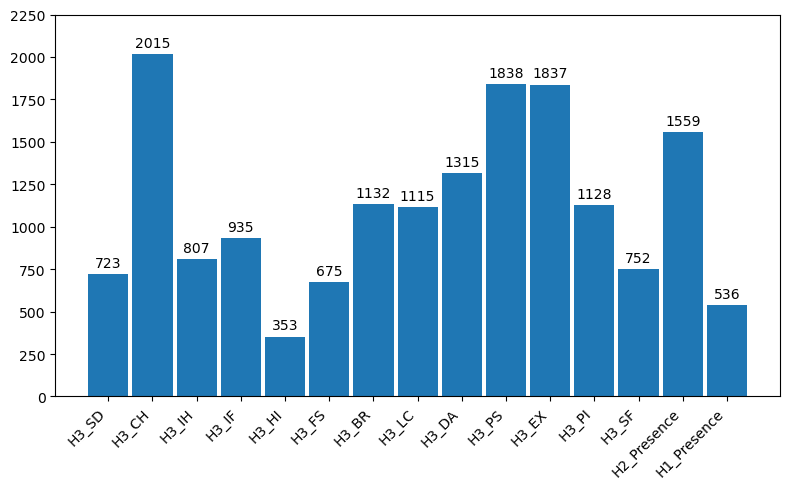

Supplement: ooaf142_Supplementary_Data [file ooaf142_supplementary_data.zip › outputClassDistribution (2).png]

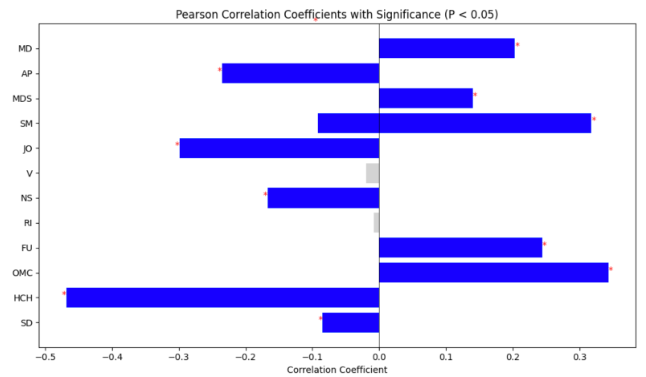

Supplement: ooaf142_Supplementary_Data [file ooaf142_supplementary_data.zip › PearsonCC (2).png]

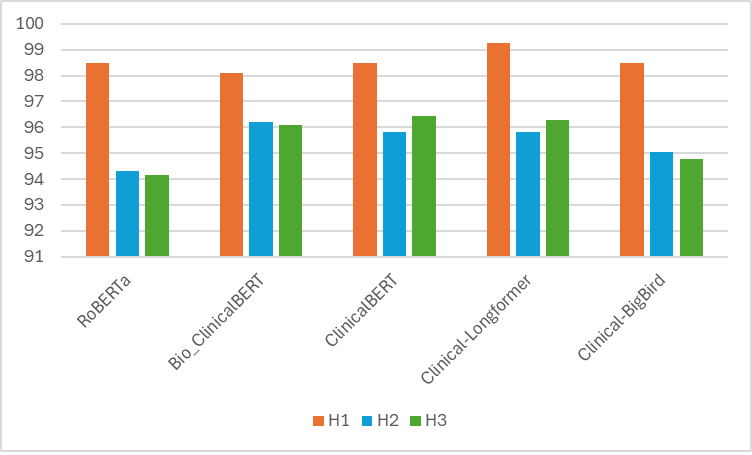

Supplement: ooaf142_Supplementary_Data [file ooaf142_supplementary_data.zip › Performance comparisons of Multilevel version (2).png]

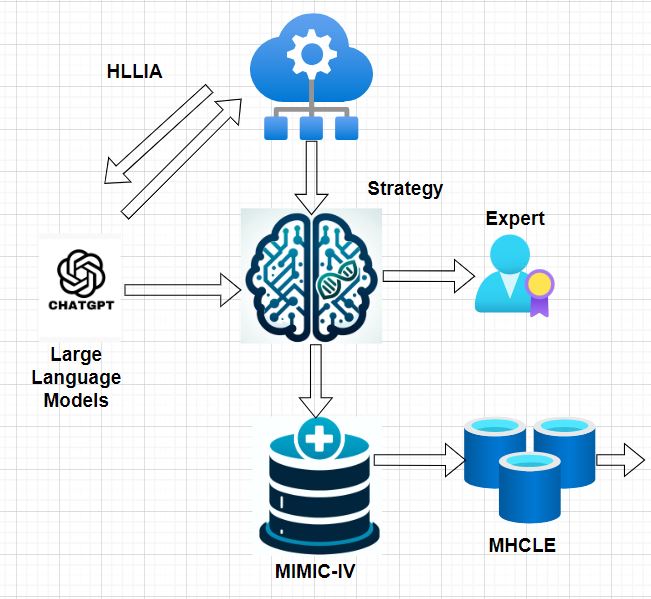

Supplement: ooaf142_Supplementary_Data [file ooaf142_supplementary_data.zip › Project1 (3).JPG]

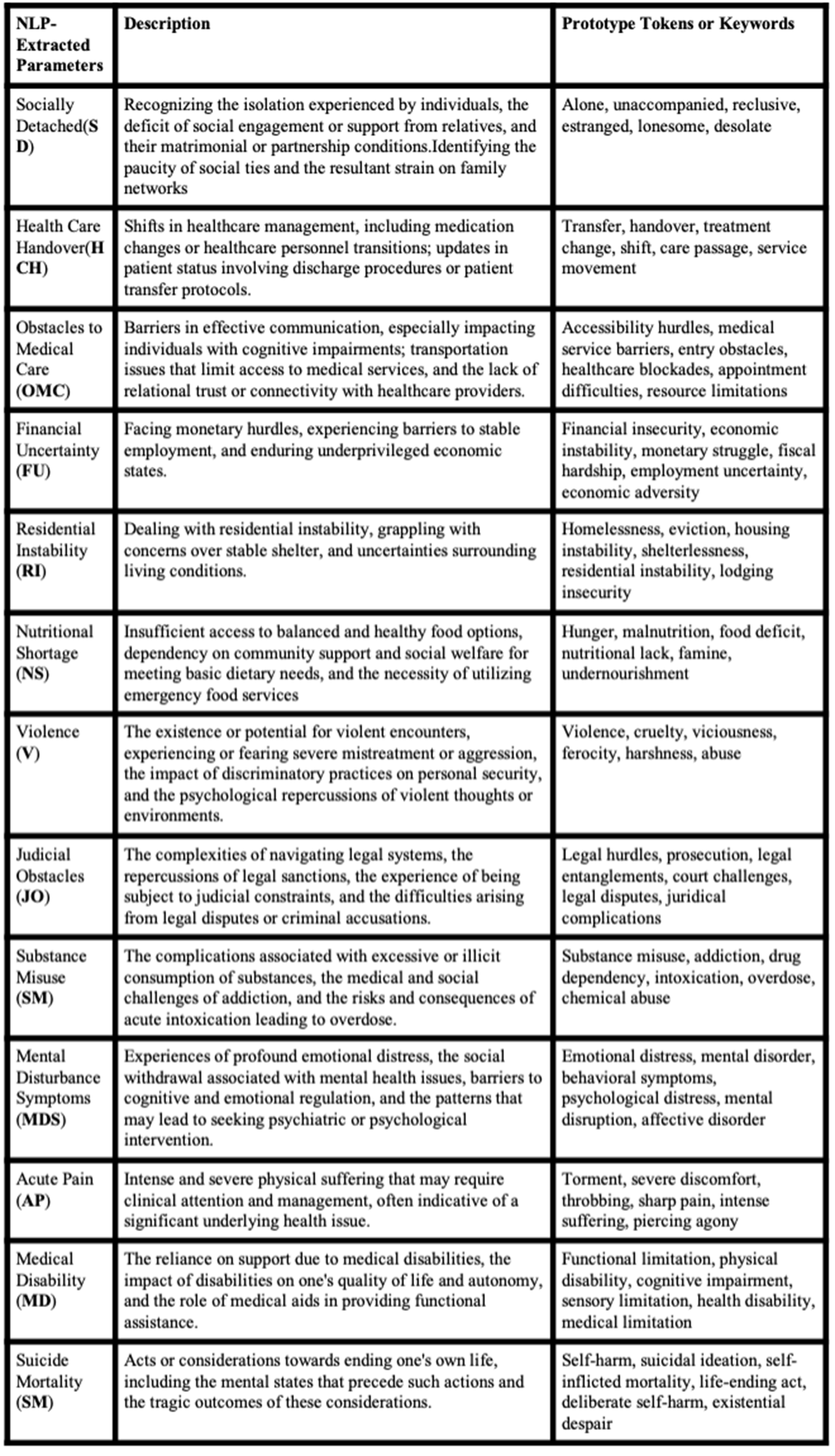

Supplement: ooaf142_Supplementary_Data [file ooaf142_supplementary_data.zip › Table_Social_determinants (3).png]
